# Increased cell proliferation in the rat anterior cingulate cortex following neonatal hypoxia: relevance to schizophrenia

**DOI:** 10.1007/s00702-012-0859-y

**Published:** 2012-07-19

**Authors:** Evelin L. Schaeffer, Franziska Kühn, Angelika Schmitt, Wagner F. Gattaz, Oliver Gruber, Thomas Schneider-Axmann, Peter Falkai, Andrea Schmitt

**Affiliations:** 1 Laboratory of Neuroscience (LIM-27), Department and Institute of Psychiatry, Faculty of Medicine, University of São Paulo, São Paulo, Brazil; 2 Department of Psychiatry and Psychotherapy, Georg-August-University, von-Siebold-Str. 5, 37075 Göttingen, Germany; 3 Department of Psychiatry and Psychotherapy, University of Würzburg, Füchsleinstr. 15, 97080 Würzburg, Germany; 4 Department of Psychiatry and Psychotherapy, Ludwig-Maximilians-University, Nußbaumstr. 7, 80336 Munich, Germany

**Keywords:** Cell proliferation, Rat, Anterior cingulate cortex, Neonatal hypoxia, Schizophrenia

## Abstract

As a consequence of obstetric complications, neonatal hypoxia has been discussed as an environmental factor in the pathophysiology of schizophrenia. However, the biological consequences of hypoxia are unclear. The neurodevelopmental hypothesis of schizophrenia suggests that the onset of abnormal brain development and neuropathology occurs perinatally, whereas symptoms of the disease appear in early adulthood. In our animal model of chronic neonatal hypoxia, we have detected behavioral alterations resembling those known from schizophrenia. Disturbances in cell proliferation possibly contribute to the pathophysiology of this disease. In the present study, we used postnatal rats to investigate cell proliferation in several brain areas following neonatal hypoxia. Rats were repeatedly exposed to hypoxia (89 % N_2_, 11 % O_2_) from postnatal day (PD) 4–8. We then evaluated cell proliferation on PD 13 and 39, respectively. These investigations were performed in the anterior cingulate cortex (ACC), caudate-putamen (CPU), dentate gyrus, and subventricular zone. Rats exposed to hypoxia exhibited increased cell proliferation in the ACC at PD 13, normalizing at PD 39. In other brain regions, no alterations have been detected. Additionally, hypoxia-treated rats showed decreased CPU volume at PD 13. The results of the present study on the one hand support the assumption of chronic hypoxia influencing transient cell proliferation in the ACC, and on the other hand reveal normalization during ageing.

## Introduction

In addition to genetic predisposition, the neurodevelopmental hypothesis of schizophrenia includes a number of environmental factors like obstetric and birth complications with hypoxia as a common factor possibly influencing the pathophysiology of the disease. The risk of schizophrenia has to be shown to increase with the severity and number of hypoxia-associated obstetric complications (Cannon et al. 1999; McNeil et al. 2000a; Dalman et al. 2001). Furthermore, twin studies revealed that fetal hypoxia was a predictor of smaller hippocampal volumes (McNeil et al. 2000b; Van Erp et al. 2002), increased ventricles and reduced cortical grey matter volume (Cannon et al. 2002), and early age of onset (Verdoux et al. 1997; Cannon et al. 2002) in schizophrenia patients. However, the consequences of hypoxia on the pathophysiology of the disease are unclear. The neurodevelopmental hypothesis of schizophrenia suggests that the onset of abnormal brain development occurs during the perinatal period, leading to symptoms of the disease in early adulthood (Weinberger 1996).

With respect to rat brain, the early postnatal period from postnatal day 4 to 8 is highly vulnerable to hypoxic damage (Ikonomidou et al. 1989). In contrast to humans, important steps of brain development in rats occur mainly postnatally and the brain growth spurt of early postnatal rats as well as overexpression of glutamatergic *N*-methyl-d-aspartate (NMDA) receptors is comparable to the third trimester in humans (Dobbing and Sands 1979). Moreover, while neuron birth and differentiation are largely completed by the end of gestation, selected regions of the brain harbor neuronal precursor cells throughout life, thus offering an opportunity to investigate stem cell proliferation postnatally. In postnatal and adult brain, new neurons continue to be generated in two specific neurogenic areas, the subgranular zone (SGZ) of the hippocampal dentate gyrus (DG) and the subventricular zone (SVZ) of the lateral ventricles (Yamashima et al. 2007). In the medial prefrontal cortex of rats inclusive anterior cingulate cortex (ACC), adult cell proliferation and its stimulation have been observed as well, but stem cells did not differentiate into neurons (Kodama et al. 2004, Madsen et al. 2005). Adult neurogenesis is a multi-step process (proliferation, differentiation, migration, targeting, and synaptic integration). Thus, from the two neurogenic areas, new neurons migrate towards their final targets in other brain areas where they differentiate and integrate with local circuits (Ehninger and Kempermann 2008). New neurons leaving the SGZ migrate into the adjacent granule cell layer of the DG (Altman and Bayer 1990; Kuhn et al. 1996). Neurons that originate in the SVZ migrate, among others, to the olfactory bulb (Pencea et al. 2001; Bédard and Parent 2004; Shapiro et al. 2006), association neocortex including prefrontal cortex (Gould et al. 1999), and striatum (Bédard and Parent 2004).

Dysregulated neurogenesis during early adulthood has been observed in some animal models of schizophrenia, implicating that postnatal neurogenesis may contribute to a part of the symptoms of this disorder. Keilhoff et al. (2004b) demonstrated enhanced adult neurogenesis in the hippocampal SGZ in a rat ketamine model of schizophrenia. Ketamine, an antagonist of *N*-methyl-d-aspartate (NMDA) receptors, has been shown to mimic behavioral and biological aspects of schizophrenia in animals (Becker et al. 2003; Bernstein et al. 2003; Keilhoff et al. 2004a). Recently, Manning et al. (2012) documented increased adult neurogenesis in the granule cell layer of the DG in phospholipase C-β1 knockout mice. Previous characterization of these mice demonstrated the presence of several schizophrenia-like endophenotypes (McOmish et al. 2008a, b).

In a previous study using a rat model of chronic neonatal hypoxia, we showed decreased NMDA receptor binding in the ACC, frontal regions, nucleus accumbens, and hippocampus of hypoxia-treated animals, as well as a deficit in prepulse inhibition of acoustic startle response (Schmitt et al. 2007; Fendt et al. 2008), which is also disrupted in schizophrenic patients and restored by antipsychotics (Braff et al. 1992; Van den Buuse et al. 2003; Kodama et al. 2004). In the present study, we used postnatal rats to investigate if there are indications of altered cell proliferation following neonatal hypoxia. We applied chronic, repeated hypoxia (11 % O_2_, 89 % N_2_) during 6 h from postnatal day 4 to 8. We then evaluated cell proliferation on postnatal days, 13 and 39, respectively. These investigations were performed in the ACC, striatum, i.e., caudate-putamen (CPU), DG, and SVZ.

## Materials and methods

### Animals and induction of neonatal hypoxia

Animal use procedures were in strict accordance with the NIH guidelines for the care and use of laboratory animals and had been approved by the local ethics committee. Forty male Sprague–Dawley rats (Animal Facility, University of Göttingen) were bred from 8 pregnant females (Charles River, Germany). Male litter mates were grown until examined on postnatal day (PD) 13 or 39. The rats were housed in standard cages under a 12 h light/dark cycle with food and water available ad libitum. Chronic, repeated hypoxia (11 % O_2_, 89 % N_2_) was imposed from PD four to eight on 20 animal pups and their mothers by placing them in an air-tight plastic chamber for a period of 6 h per day. The 20 other pups were subjected to identical handling conditions and were placed in identical chambers but with regular oxygen concentrations (21 % O_2_ = normoxia; control animals) (Schmitt et al. 2007; Fendt et al. 2008; Sommer et al. 2010). During hypoxia, mothers were able to care for their pups.

### BrdU injection and brain tissue collection

On PD 11, 20 hypoxia-treated (mean weight: 21.1 ± 1.54 g) and 20 control rats (mean weight: 25.5 ± 1.42 g) received intraperitoneal (i.p.) injection of 5-bromo-2-deoxyuridine (BrdU; Sigma, Taufkirchen, Germany) four times every 2 h (75 mg/kg body weight, dissolved in 0.9 % NaCl), for labeling the newly dividing cells. BrdU, a thymidine analogue, is incorporated into the DNA of dividing cells during the S-Phase and is detected using a specific, monoclonal antibody (Gratzner 1982). To investigate the effect of postnatal hypoxia on stem cell proliferation, 10 hypoxia-treated and 10 control rats were killed 2 days after BrdU injection (PD 13). Additionally, 10 hypoxia-treated and 10 control rats were allowed to survive 28 days after BrdU injection (PD 39). Rats were anesthetized with i.p. injection of ketamine (100 mg/kg bw)/xylazine (5 mg/kg bw) and killed by transcardial perfusion with Tyrode’s solution followed by fixation with 4 % paraformaldehyde in Sörensen buffer 0.2 M, pH 7.6 (NaH_2_PO_4_ and Na_2_HPO_4_). The brains were dissected and postfixed in 4 % paraformaldehyde for 2 h and subsequently immersed in 15 % sucrose solution overnight at 4 °C. The brain tissue was then stored until use at −80 °C.

### BrdU immunohistochemistry

The brains were cut by cryostat in the coronal plane from frontal pole until the end of hippocampus into 60-μm-thick adjacent serial sections. Every sixth free-floating section was processed for immunohistochemical staining of incorporated BrdU. Brain sections were washed in TRIS-buffered saline (TBS, pH 7.5) at room temperature (RT). Next, the sections were incubated with 0.6 % H_2_O_2_ for 30 min at RT to eliminate endogenous peroxidase activity. After washing in TBS, DNA was denaturated with 50 % formamide/2× saline sodium citrate (SSC; pH 7.0) for 2 h at 65 °C followed by acid (2N HCl). After washing in TBS, the sections were then incubated with 2N HCl for 30 min at 37 °C. Sections were washed in 100 mM borate buffer (pH 8.5) for 10 min at RT to neutralize the acid. After washing in TBS, the sections were incubated for 60 min at RT with blocking solution A [2 % bovine serum albumin (BSA) and 5 % normal goat serum (NGS) in 0.25 % Triton X-100-TBS]. They were then incubated overnight at 4 °C with 1:500 anti-BrdU mouse monoclonal (Roche, Mannheim, Germany) diluted in blocking solution A. After washing in TBS, the sections were incubated for 60 min at RT with 1:400 biotinylated anti-mouse (Vectastain ABC Kit, Linaris, Wertheim, Germany) diluted in blocking solution B (2 % BSA and 2 % NGS in 0.25 % Triton X-100-TBS). Sections were washed in TBS and subsequently incubated with avidin-biotinylated horseradish peroxidase complex (Vectastain ABC Kit) for 90 min at RT. After washing in TBS, sections were treated with diaminobenzidine (DAB; Vectastain ABC Kit) for 10 min at RT and subsequently washed in TBS. Finally, the sections were mounted on slides and coverslipped.

### Stereological analysis

**Fig. 1:**
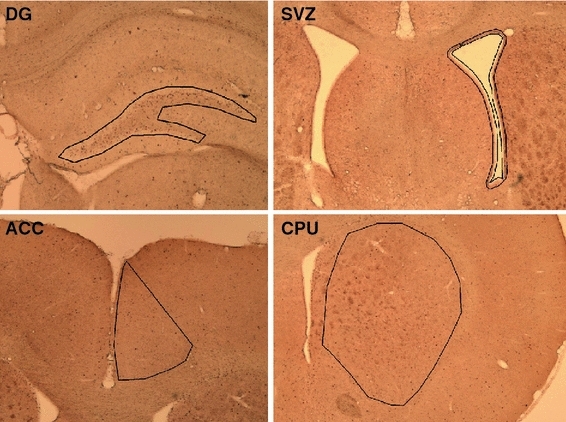
Delineation of areas of interest in the ACC, CPU, SVZ and DG

**Fig. 2:**
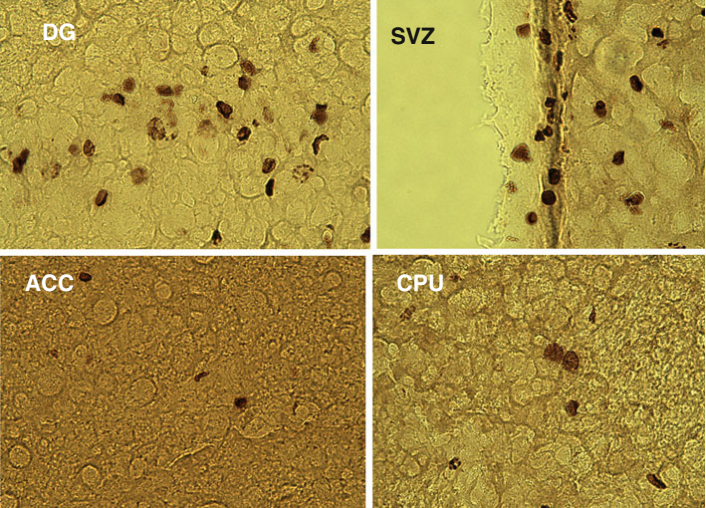
Sections of BrdU-stained cells at 28 days after injection in the ACC, CPU, SVZ and DG at ×50 magnification

Regions were delineated according to the atlas of Paxinos and Watson (1986) (Fig. 1). BrdU-labeled cells were counted in serial sections in the ACC, CPU, DG, and SVZ (Fig. 2) using a stereological workstation, consisting of a modified light microscope (BX50; Olympus, Tokyo, Japan), Olympus Uplan Apo objectives (1.5×, 20×, 50×oil, 100×oil), motorized specimen stage for automatic sampling, electronic microcator, CCD color video camera, PC with frame grabber board, stereology software (StereoInvestigator; MicroBrightField, Williston, USA) and a 17-in. monitor. Boundaries of the regions of interest (ACC, CPU, DG, SVZ) (Fig. 1) were traced on video images displayed on the computer screen and total volumes of the delineated structures were calculated according to the Cavalieri principle (Schmitz and Hof 2005). With respect to the CPU, the total structure was delineated, while in the other regions we delineated areas of interest (Fig. 1). Total numbers of BrdU-positive cells were estimated using the optical disector (West et al. 1991; Schmitz 1998; Schmitz and Hof 2000, 2005). We performed a count of BrdU-labeled cells that came into focus within unbiased virtual counting spaces (Sterio 1984; Gundersen et al. 1988) distributed in a systematic-random fashion throughout the different regions of interest. Total estimated numbers of BrdU-labeled cells were calculated by the area that was sampled on each section of known thickness and the distance between the corresponding surfaces of the adjacent systematically sampled sections according to the Cavalieri method (Gundersen et al. 1988; West 1999).

### Statistical analysis

Statistical analysis was performed using SPSS 17 software. The body weight gain data were analyzed by one-way ANOVA. Results of Kolmogorov–Smirnov tests showed normal distribution of cell number and structure volume data. Thus, comparisons between the hypoxia-treated rats and controls were performed using generalized linear model multivariate analysis (MANOVA), with treatment such as fixed factor and body weight as covariate. In all analyses, differences were considered statistically significant if *p* value was smaller than 0.05. All data are expressed as mean ± SD.

## Results

### Postnatal day 13

**Fig. 3:**
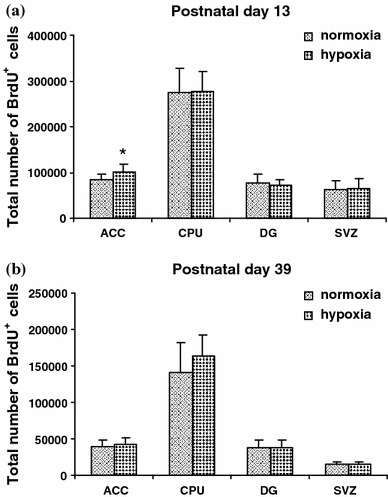
**a** Stereological analysis of cell proliferation in the anterior cingulate cortex (*ACC*), caudate-putamen (*CPU*), dentate gyrus (*DG*), and subventricular zone (*SVZ*), as measured in rats injected with BrdU and killed 2 days later. Data are reported as mean ± SD (*n* = 10 per group). The total estimated number of BrdU-positive cells was significantly increased in the ACC of hypoxia-treated rats when compared with normoxia (control) animals at postnatal day 13. No differences were observed in the number of BrdU-labeled cells between hypoxia-treated and control rats in the CPU, DG, and SVZ. *P < 0.05, MANOVA. BrdU, 5-bromo-2-deoxyuridine. **b** Stereological analysis of survival of newly generated cells in the anterior cingulate cortex (*ACC*), caudate-putamen (*CPU*), dentate gyrus (*DG*), and subventricular zone (*SVZ*), as measured in rats injected with BrdU and killed 28 days later. Data are reported as mean ± SD (*n* = 10 per group). No differences were observed in the total estimated number of BrdU-positive cells between hypoxia-treated rats and normoxia (control) animals in the ACC, CPU, DG, and SVZ at postnatal day 39. BrdU, 5-bromo-2-deoxyuridine

**Fig. 4:**
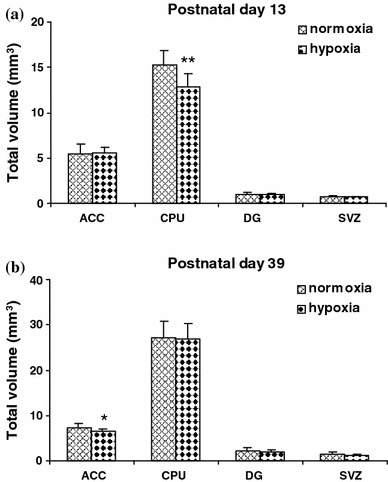
**a** Volumes of the areas of interest anterior cingulate cortex (*ACC*), caudate-putamen (*CPU*), dentate gyrus (*DG*), and subventricular zone (*SVZ*) measured in sections used for stereological counts of BrdU-positive cells in rats injected with BrdU and killed 2 days later. Data are reported as mean mm^3^ ± SD (*n* = 10 per group). The total CPU volume was significantly decreased in hypoxia-treated rats when compared with normoxia (control) animals at postnatal day 13. No differences were observed in ACC, DG, and SVZ volumes between hypoxia-treated and control rats at postnatal day 13. **P < 0.001, MANOVA. BrdU, 5-bromo-2-deoxyuridine. **b** Volumes of the areas of interest anterior cingulate cortex (*ACC*), caudate-putamen (*CPU*), dentate gyrus (*DG*), and subventricular zone (*SVZ*) measured in sections used for stereological counts of BrdU-positive cells in rats injected with BrdU and killed 28 days later. Data are reported as mean mm^3^ ± SD (*n* = 10 per group). The total ACC volume was significantly decreased in hypoxia-treated rats when compared with normoxia (control) animals at postnatal day 39. No differences were observed in CPU, DG, and SVZ volumes between hypoxia-treated and control rats at postnatal day 39. *P < 0.05, MANOVA. BrdU, 5-bromo-2-deoxyuridine

On PD 13, the hypoxia-treated rats showed lower body weight than controls (hypoxia: 25.26 ± 2.07 g vs. controls: 29.01 ± 1.83 g; ANOVA: *F* = 18.5, *p* < 0.005). Stereological analysis revealed that the hypoxia-treated animals had significantly higher absolute numbers of BrdU-labeled cells in the ACC when compared with controls (MANOVA: *F* = 6.0, *p* = 0.025; Fig. 3a). No significant differences between the hypoxia-treated rats and the controls were found in absolute numbers of BrdU-labeled cells in the other brain regions investigated, namely, the CPU, DG and SVZ (all MANOVA: *F* = 0, 0.6, 0 respectively, *p* > 0.1; Fig. 3a). Regarding volumes of the regions of interest, the hypoxia-treated animals showed a significant decrease in the total volume of the CPU when compared with controls (MANOVA: *F* = 12.4, *p* = 0.002; Fig. 4a). We did not detect any difference between the hypoxia-treated rats and the controls with respect to the total volumes in the areas of interest ACC, DG and SVZ (all MANOVA: *F* = 0, 0.1, 0.2 respectively, *p* > 0.5; Fig. 4a).

### Postnatal day 39

On PD 39, the hypoxia-treated rats showed significantly lower body weight than controls (hypoxia: 179.0 ± 16.63 g vs. controls: 197.5 ± 20.17 g; ANOVA: *F* = 5.0, *p* = 0.038). Stereological analysis showed that there were no significant differences between the hypoxia-treated animals and the controls with respect to the absolute numbers of BrdU-labeled cells in the investigated brain regions, i.e., ACC, CPU, DG and SVZ (all MANOVA: *F* = 0.6, 2.0, 0, 0.2 respectively, *p* > 0.1; Fig. 3b). With respect to volumes of the regions of interest, the hypoxia-treated animals showed a significant decrease in the total volume of the ACC when compared with controls (MANOVA: *F* = 5.0, *p* = 0.044; Fig. 4b). However, multivariate analysis revealed an effect of weight on the total volume of the ACC in the hypoxia-treated rats (MANOVA: *F* = 13.6, *p* = 0.002). We did not detect any difference between the hypoxia-treated rats and the controls with respect to the total volume in the CPU, DG and SVZ (all MANOVA: *F* = 0.1, 2.3, 1.5 respectively, *p* > 0.1; Fig. 4b).

## Discussion

The main finding of this study was that cell proliferation was significantly increased by 20 % in the ACC of hypoxia-treated rats at PD 13 (5 days after the cessation of hypoxia). The study also showed that total volume of the CPU was significantly decreased by 16 % in hypoxia-treated rats at PD 13. At PD 39 in the ACC, we did not find differences in the number of proliferating cells, but the volume of this region of interest was lower in the hypoxia animals, possibly concealing alterations in the hypoxia group. The unchanged volumes of the other regions of interest, where proliferating cells were counted, point to the validity of our comparison between study groups.

The finding of increased cell proliferation at PND 13 in our neonatal rat model of chronic hypoxia is only partially in line with other studies in animal models of neonatal hypoxia, since we have found increased cell proliferation but in a different brain area from those reported in other models. In schizophrenia, a post-mortem study showed decreased cell proliferation in the dentate gyrus (Reif et al. 2006) and it has been hypothesized that neonatal hypoxia may contribute to these findings. Our animal model of chronic neonatal hypoxia does not support the hypothesis of environmental factors contributing to altered cell proliferation in the hippocampus. However, in a model of brief hypoxia in newborn rats, where pups were exposed within 24 h after birth to 100 % nitrogen (N_2_) for 5 or 20 min at 36 °C, the number of proliferative cells was found to be increased in the SVZ and hippocampal DG of 21-day-old pups (Daval et al. 2004; Pourié et al. 2006; Blaise et al. 2009). In a model of transient hypoxia in mice, which were reared in a low-oxygen environment (9.5–10.5 % O_2_) from PD 3 to 11, a marked increase in cell proliferation was observed in the SVZ at PD 18, 7 days after the cessation of hypoxia (Fagel et al. 2006). In our animal model of 6 h hypoxia per day from PD 4–8 we may fail to show alterations in the hippocampus or SVZ due to possibly weaker effects of the applied hypoxia. Moreover, our findings of increased cell proliferation only in the ACC following chronic neonatal hypoxia in rats are not in accordance with findings in two animal models of schizophrenia. Keilhoff et al. (2004b) reported increased number of proliferative cells in the hippocampal SGZ in ketamine-treated rats aged 2 months, an animal model of schizophrenia. Ketamine has been shown to mimic behavioral and biological correlates of schizophrenia in animals (Becker et al. 2003; Bernstein et al. 2003; Keilhoff et al. 2004a). Manning et al. (2012) documented increased number of proliferative cells in the granule cell layer of the DG in phospholipase C-β1 knockout mice aged 2 months, which show several schizophrenia-like endophenotypes. To our knowledge, the present study is the first to demonstrate increased cell proliferation in the ACC in an animal model of neonatal hypoxia, a brain area that appears to be critically involved in the pathophysiology of schizophrenia (Job et al. 2002; Fornito et al. 2008; Witthaus et al. 2009; Lui et al. 2009). The ACC has been shown to be involved in other environmental risk factors of schizophrenia such as urban upbringing (Lederbogen et al. 2011). In post-mortem studies of schizophrenia patients, in this region neuronal density and neuronal size has been shown to be decreased (Benes et al. 1986; Benes and Bird 1987).

The mechanisms by which neonatal hypoxia may induce cell proliferation remain to be elucidated. Keilhoff et al. (2004b) hypothesize that in their animal model of schizophrenia, ketamine may evoke its stimulating effect on hippocampal neurogenesis by blocking the NMDA receptor directly via reduction of c-Fos and c-Jun expression, leading to a depression of the AP1 transcription factor complex, and/or via a reduced NO production or an enhanced serotonergic activity. In our animal model of chronic neonatal hypoxia, at PD 13, rats exhibited decreased NMDA receptor binding in the ACC, frontal regions, nucleus accumbens, and hippocampus along with a compensatory upregulation of NR1 mRNA (Schmitt et al. 2007), which suggests an involvement of the NMDA receptor in the pathophysiology of hypoxia-induced alterations, including enhanced cell proliferation.

Our data of reduced striatum (CPU) volume at PD 13 is consistent with findings of volumetric deficits in multiple brain regions, including the striatum (caudate nuclei, nucleus accumbens, putamen) in never-medicated, first-episode schizophrenic patients (Corson et al. 1999; Lieberman et al. 2001; Job et al. 2002; Chua et al. 2007; Glenthoj et al. 2007; Kaspárek et al. 2007; Fornito et al. 2008; Witthaus et al. 2009; Lui et al. 2009; Watson et al. 2012; Asami et al. 2012), when many confounders such as age of onset, duration of illness and medication are reduced or absent. However, except the volume of CPU we did not measure volumes or neuronal cell numbers in other brain regions of hypoxia-treated rats and hypoxia may cause apoptosis-induced cell loss. Indeed, combined hypoxia–ischemia has been shown to induce aberrant DNA synthesis along with apoptosis as revealed by BrdU and transferase-mediated biotinylated UTP nick end labeling (TUNEL) double-staining (Kuan et al. 2004; Burns et al. 2007). Thus, although these damaged neurons enter the S-phase, they might die due to apoptosis without differentiating into new neurons. Aberrant activation of the cell cycle machinery is known to induce apoptosis and delayed neuronal death after hypoxia–ischemia (Katchanow et al. 2001). However, in our study the ischemic component has not been applied. But a previous 20 min induction of severe neonatal hypoxia revealed delayed cell death in the hippocampal region CA1, reflecting apoptosis followed by increased neurogenesis (Daval et al. 2004). A 5-min hypoxia period again did not induce apoptosis (Blaise et al. 2009; Pourié et al. 2006). Hence the severity of hypoxia seems to influence the incidence of apoptosis which should be further investigated in our animal model.

Since we did not investigate the development of proliferating cells into neurons or glial cells further studies are needed to address this issue. One of these is, e.g., the study of Fagel and co-workers reporting that chronic perinatal hypoxia in mice leads to an increase of astroglial cell proliferation in the subventricular zone. On day 28, in the cerebral cortex the number of BrdU-positive cells increased and differentiated into oligodendrocytes (45 %) and astrocytes (35 %) while only 10 % were identified as neurons. However, in the neocortex, twice as many BrdU-labeled cells expressed neuronal markers compared to controls (Fagel et al. 2006). In addition, survival of immature neurons is known to be affected by higher BrdU concentrations over 50 mg/kg such as applied by ourselves. Moreover, despite the selection of BrdU-positive cells along morphological criteria like cell size and shape, counting of stained microglia cannot be excluded and the findings of increased cell proliferation in the ACC might be influenced by microglial reaction. We did not measure behavioral alterations like deficits in PPI after neonatal hypoxia and behavior might be altered by BrdU-induced cell death of hippocampal neurons. However, stress induced by behavioral testing possibly also influences cell proliferation (Jayatissa and Henningsen 2010) for which reason we decided against performing PPI measurements. Besides, we did not find PPI differences in this young age group before puberty in our previous study (Fendt et al. 2008). Further investigations should include measurements of cell proliferation in rats exposed to neonatal hypoxia during adulthood, when symptoms of the disease occur.

In summary, we have shown alterations in cell proliferation and volume of the CPU after repeated hypoxia in brain regions involved in the pathophysiology of schizophrenia. The results of the present study, together with those in previous studies showing the association between chronic neonatal hypoxia and schizophrenia-related behavioral alterations in postnatal rats (Schmitt et al. 2007; Fendt et al. 2008), support the hypothesis that neonatal hypoxic brain injury may be one of several causal factors for abnormalities in behavioral development and subsequent schizophrenia.
